# Association of Computed Tomographic Screening Promotion With Lung Cancer Overdiagnosis Among Asian Women

**DOI:** 10.1001/jamainternmed.2021.7769

**Published:** 2022-01-18

**Authors:** Wayne Gao, Chi Pang Wen, Allison Wu, H. Gilbert Welch

**Affiliations:** 1 College of Public Health, Taipei Medical University, Taipei City, Taiwan; 2 Institute of Population Health Science, National Health Research Institutes/China Medical University Hospital, Zhunan, Taiwan; 3 Center for Surgery and Public Health, Brigham and Women’s Hospital, Boston, Massachusetts

## Abstract

**Importance:**

As smoking continues to decline in many developed countries, the proportion of lung cancers in nonsmokers will rise. This shift may create substantial pressure to further expand lung cancer screening to lower-risk groups.

**Objective:**

To determine the association of lung cancer incidence with the promotion of screening in a largely nonsmoking population.

**Design, Setting, and Participants:**

This population-based ecological cohort study of stage-specific lung cancer incidence used the Taiwan Cancer Registry to identify women diagnosed with lung cancer from January 1, 2004, to December 31, 2018. Smoking prevalence among Taiwanese women has been less than 5% since 1980. Data were analyzed from February 13, 2020, to November 10, 2021.

**Exposures:**

Low-dose computed tomography (LDCT) screening for lung cancer, initiated during the early 2000s.

**Main Outcomes and Measures:**

Change in stage-specific lung cancer incidence. An effective cancer screening program will not only increase the incidence of early-stage cancer but also decrease the incidence of cancer presenting at a late stage.

**Results:**

A total of 57 898 women were diagnosed with lung cancer in a population of approximately 12 million Taiwanese women. After the introduction of LDCT screening, the incidence of early-stage (stages 0-I) lung cancer in women increased more than 6-fold, from 2.3 to 14.4 per 100 000 population (absolute difference, 12.1 [95% CI, 11.3-12.8]) from 2004 to 2018. There was no change, however, in the incidence of late-stage (stages II-IV) lung cancer, from 18.7 to 19.3 per 100 000 (absolute difference, 0.6 [95% CI, –0.5 to 1.7]). Because the additional 12.1 per 100 000 early-stage cancers were not accompanied by a concomitant decline in late-stage cancers, virtually all the additional cancers detected represent overdiagnosis. Despite stable mortality, 5-year survival more than doubled from 2004 to 2013, from 18% to 40%, which is arguably the highest lung cancer survival rate in the world.

**Conclusions and Relevance:**

This population-based ecological cohort study found that low-dose computed tomographic screening of mostly nonsmoking Asian women was associated with considerable lung cancer overdiagnosis. Five-year survival is biased by the increased LDCT detection of indolent early-stage lung cancers. Unless randomized trials can demonstrate some value to low-risk groups, LDCT screening should remain targeted only to heavy smokers.

## Key Points

**Question:**

What happens to lung cancer incidence when lung cancer screening is promoted to a population with a smoking prevalence of less than 5%?

**Findings:**

In this population-based ecological cohort study of approximately 12 million Taiwanese women, the promotion of lung cancer screening was associated with a 6-fold increase in the incidence of early-stage (stages 0-I) lung cancer from 2004 to 2018, whereas there was no change in the incidence of late-stage (stages II-IV) lung cancer. Five-year survival rates have more than doubled to 40% despite stable lung cancer mortality.

**Meaning:**

Lung cancer screening in a largely nonsmoking population was associated with considerable overdiagnosis and spuriously high 5-year survival rates.

## Teaser

This cohort study investigates whether opportunistic screening for lung cancer among Taiwanese women who rarely smoke is associated with overdiagnosis of lung cancer.

## Introduction

In March 2021, the US Preventive Services Task Force expanded eligibility for lung cancer screening to individuals with less smoking exposure.^1^ Their original recommendation in 2013 reflected the criterion used by the National Lung Screening Trial: exposure of 30 or more pack-years.^2^ Their new recommendation expanding the criterion to 20 or more pack-years was informed by modeling studies and the recent European trial in which one-quarter of those randomized had less than 30 pack-years of exposure.^3^

According to estimates from the International Agency for Research on Cancer for the year 2000, 85% of lung cancer in men and 47% of lung cancer in women is attributable to smoking.^4^ However, as cigarette smoking continues to decline in the US,^5^ so too will the proportion of lung cancers attributable to smoking. Conversely, the proportion of lung cancers occurring in those who have never smoked cigarettes will rise, as has already been reported by some hospitals.^6^ This shift may create substantial pressure to further expand lung cancer screening to nonsmokers in the future.

Once expansive screening criteria are established, it is often difficult to reverse or narrow them.^7^ Opportunistic lung cancer screening is being popularized throughout Asia; China,^8,9^ Japan,^10,11^ South Korea,^12,13^ and Taiwan^14^ have hospital-based programs that routinely include nonsmokers. In this report, we consider the association of opportunistic screening in the population of Taiwanese women—95% of whom have never smoked—with an unintended adverse effect: lung cancer overdiagnosis.

In Taiwan, low-dose computed tomography (LDCT) screening for lung cancer is currently not covered by the National Health Insurance (NHI), a single-payer, fee-for-service compulsory health insurance program with a global budget that covers 99.7% of the population.^15^ There have been strong calls for the NHI to cover lung cancer screening, however, by both health care professionals and celebrities who believe their lives have been saved by screening.^16,17^ Although Taiwanese hospitals and physicians cannot directly advertise medical services, LDCT screening has been promoted in the media and on hospital websites. Screening has been priced low (approximately $150-$230) and has been offered as a free charitable service to selected groups (eg, teachers, firefighters, middle- to low-income women, and indigenous people). Hospitals generate revenue from subsequent follow-up testing, biopsies, and surgical procedures covered by the NHI.^18^

**Figure 1.**
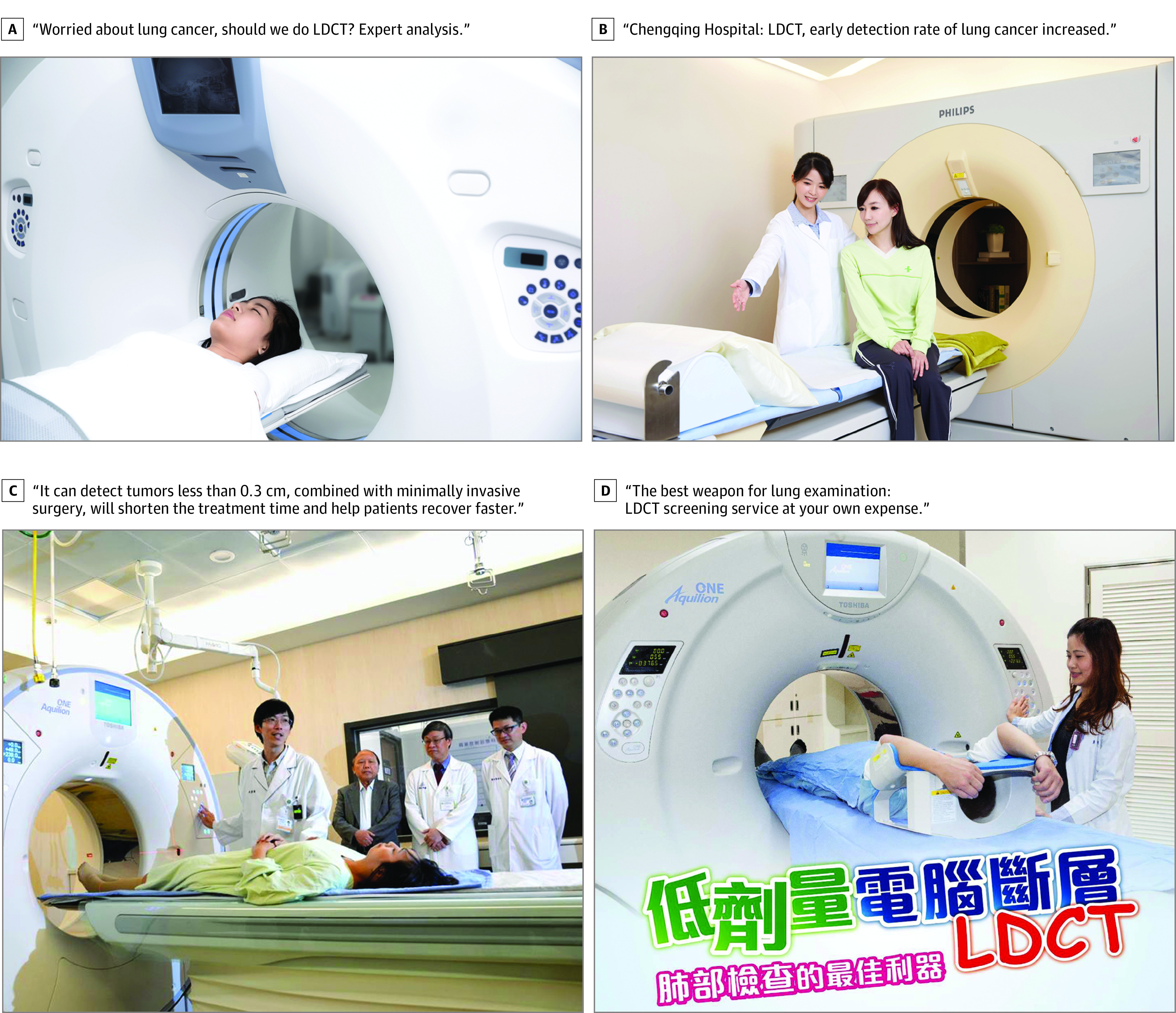
Taiwanese Lung Cancer Screening Promotions Featuring Young Women LDCT indicates low-dose computed tomography. A, Reprinted with permission from Mr Suwannaphoom and copyright holder.^19^ B, Reprinted with permission from the *InfoTimes*.^20^ C, Reprinted with permission from Ms Yao and copyright holder.^21^ D, Reprinted with permission from Department of Medical Imaging and Intervention Chang Gueng Memorial Hospital, Taiwan.^22^

Taiwanese women are often featured in LDCT promotion (Figure 1).^19,20,21,22^ Images of young women entering recently purchased high-precision CT scanners are accompanied by dramatic language:

- Avoid the tragedy of sudden death from terminal lung cancer like the stars (celebrities). People who have never done LDCT, especially women, should do it now*.*^23^
- Women are genetically more fragile and cannot easily repair diseased cells, and they should be regularly checked [with LDCT].^24^

**Figure 2.**
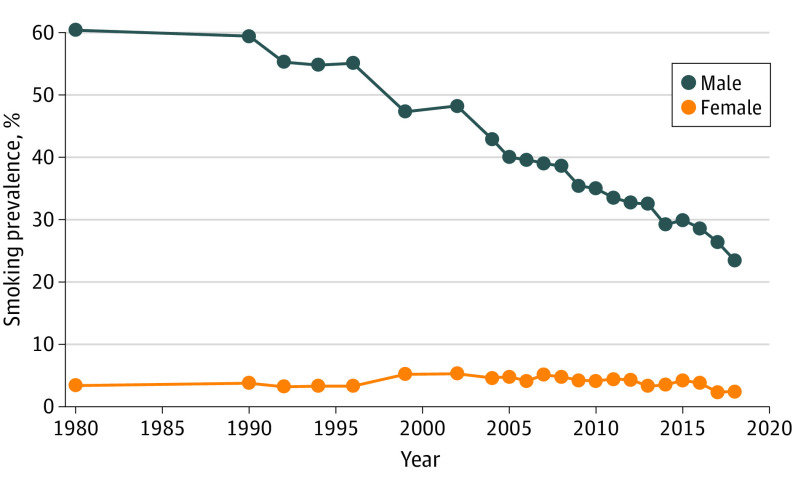
Self-reported Smoking Prevalence Among Taiwanese Adults by Sex, 1980-2018 Data are from the Adult Smoking Behaviors Survey 2018, Health Promotion Administration, Taiwan.^25^ Adult indicates 18 years or older. Before 1997, a smoker was defined as smoking on average 3 or more cigarettes per day; after 1997, a smoker was defined as smoking every day or smoking sometimes in the last 30 days.

The promotion targeting women is particularly noteworthy because Taiwanese women rarely smoke; smoking prevalence among women has been less than 5% since 1980 (Figure 2).^25^

## Methods

This population-based ecological cohort study followed the Strengthening the Reporting of Observational Studies in Epidemiology (STROBE) reporting guideline. The study used the aggregated and deidentified database of a national cancer registry, for which institutional review board approval and informed consent are not required in Taiwan.

To examine the possibility of overdiagnosis after LDCT promotion targeting women, we calculated stage-specific lung cancer incidence using data from the Taiwan National Cancer Registry.^26^ Founded in 1979, the Taiwan National Cancer Registry is a population-based cancer registry that has met the quality criteria for inclusion in the worldwide surveillance of trends in cancer survival (CONCORD 2 and 3).^27,28^ Since 2003, annual data completeness has exceeded 94% (range across years, 94.3%-98.4%), and morphological verification has been obtained in more than 94% for all sites excluding the liver (range across years, 94.4%-97.6%).^29^

Specifically, we sought evidence on the 2 fundamental prerequisites for an effective screening program: (1) increased early-stage incidence, demonstrating that screening detects cancer early, and (2) decreased late-stage incidence, demonstrating that screening leads to a reduction in the presentation of advanced cancer. In the setting of stable true cancer occurrence, rising early-stage incidence not followed by a concomitant decline in late-stage incidence is pathognomonic of overdiagnosis.

Stage-specific counts of lung cancer were available beginning in 2004. This approximates the time that LDCT screening first appeared in Taiwan; hospitals began to promote the service in the mid-2000s, and the number of CT scanners registered to the Taiwan Atomic Energy Council grew from 357 in 2003 to 623 in 2019.^30,31^

We constructed 2 broad stage categories: early (stages 0 [carcinoma in situ] and I [tumor size ≤4 cm and no nodal involvement]) and late (stages II through IV [tumor size >4 cm or nodal involvement or metastasis to distant sites]). Using the stage-specific counts, we calculated the stage distribution in each year from January 1, 2004, to December 31, 2018. We then multiplied data on the stage distribution and overall incidence to calculate stage-specific incidence for each year (eMethods 1 in the Supplement). For example, in 2011, the lung cancer stage distribution in women was 20% early stage and 80% late stage, and overall incidence was approximately 25 per 100 000 population. Accordingly, early-stage incidence was 5 per 100 000 population and late-stage incidence was 20 per 100 000 population. All incidence data were age adjusted to the 2000 world standard population.

Although there is no perfect method to estimate overdiagnosis, we believe it is important to provide some sense of the number of women affected. In eMethods 2 in the Supplement, we detail 2 simple approaches—excess early-stage incidence and excess early-stage counts—used to estimate the number of women overdiagnosed. Both assume that there was no overdiagnosis in 2004 and prior years, that there is no overdiagnosis of late-stage cancer, and that true lung cancer occurrence is stable; that is, there is no change in the underlying rate of clinically meaningful lung cancer.

We believe these assumptions bias our estimates downward. Some overdiagnosis in 2004 and prior years undoubtedly occurred, and there may be some overdiagnosis of stage II lung cancer, particularly in elderly women. More importantly, true lung cancer occurrence in Taiwanese women is likely to be declining. Although there has been little change in smoking prevalence among women, smoking has declined sharply among men (from >60% to <25%). Thus, women’s exposure to secondhand smoke has been declining, which would decrease the true occurrence of lung cancer. Furthermore, there has also been a general improvement in ambient air quality in Taiwan during the past few decades and a substantial decline in the use of wood or coal in cooking and heating (eFigure 1 in the Supplement). The reduction of these risk factors would only further decrease the true occurrence of lung cancer.

### Statistical Analysis

We obtained data on female standardized lung cancer mortality from 2018 Cause of Death Statistics published by the Ministry of Health and Welfare.^32^ To evaluate the outcome of changing diagnosis on apparent survival conditioned on being a case, we also examined 5-year survival rates using data from the Taiwan National Cancer Registry.^33^ For context, we also examined 5-year survival data for women with lung cancer in other countries using data from the International Agency for Research on Cancer and the Surveillance, Epidemiology, and End Results program in the US.^34,35^ Confidence intervals were obtained using Stata, version 11.2 (StataCorp LLC). Data were analyzed from February 13, 2020, to November 10, 2021.

## Results

### Epidemiologic Signature: Incidence and Mortality

**Figure 3.**
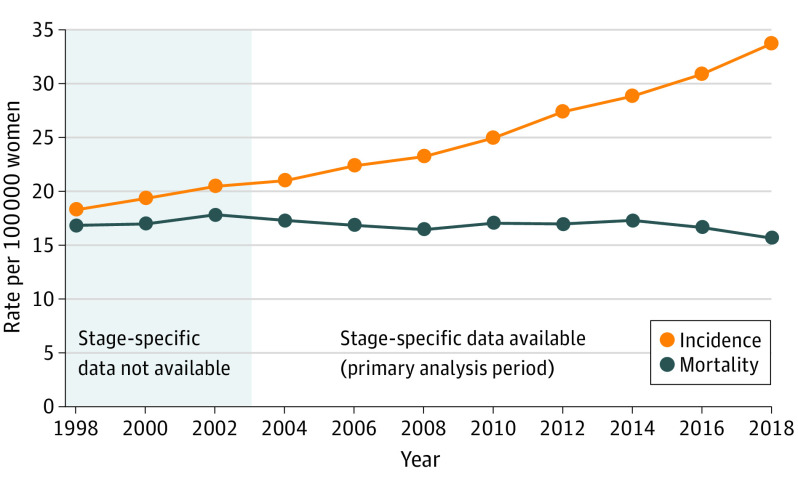
Lung Cancer Incidence and Mortality in Taiwanese Women, 1998-2018 From the Taiwan National Cancer Registry^26^ and the Ministry of Health and Welfare, Taiwan.^32^

Figure 3 illustrates lung cancer incidence and mortality trends for Taiwanese women. The epidemiologic signature of rising incidence and stable mortality suggests overdiagnosis. Figure 3 also shows that incidence and mortality were both stable during the 5 years before 2004, providing evidence that our primary analysis period (for which stage-specific data are available) largely captures the onset of LDCT screening.

### Primary Analysis: Stage-Specific Incidence

**Figure 4.**
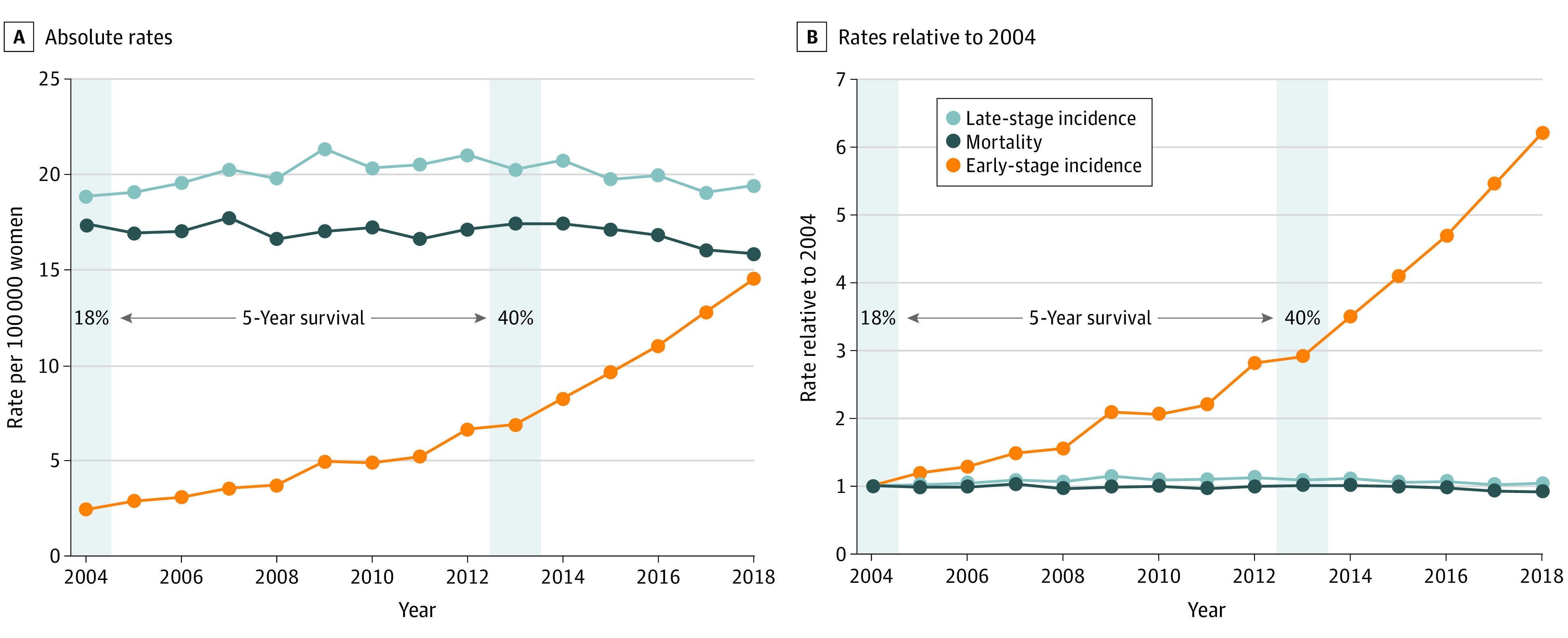
Stage-Specific Lung Cancer Incidence and Mortality in Taiwanese Women, 2004-2018 Early stage indicates stages 0 to I; late stage, stages II to IV. Absolute rates are age adjusted to the 2000 world standard population; in rates relative to 2004, 1 denotes no change. Gray shading indicates lung cancer 5-year survival for all women diagnosed with lung cancer in 2004 and 2013, the most recent year with 5 years of follow-up.

In a population of approximately 12 million women, 57 898 women were diagnosed with lung cancer from 2004 to 2018 (median age at diagnosis fell from 68 to 65 years). Our findings on stage-specific incidence are illustrated in Figure 4. Figure 4A shows the age-adjusted absolute rates of stage-specific incidence and mortality, whereas Figure 4B shows the rates relative to the base year of 2004. After the introduction of LDCT screening in Taiwanese women, we found the expected increase in rates of early-stage disease. From 2004 to 2018, their incidence of early-stage (stages 0-I) lung cancer increased more than 6-fold, from 2.3 to 14.4 per 100 000 population (absolute difference, 12.1 [95% CI, 11.3-12.8]). However, we did not observe the expected decrease in late-stage disease. The incidence of late-stage (stages II-IV) lung cancer was unchanged from 2004 to 2018, from 18.7 to 19.3 per 100 000 population (absolute difference, 0.6 [95% CI, –0.5 to 1.7]).

This combination of findings, an additional 12.1 early-stage cancers per 100 000 population and no reduction in late-stage cancers, is strongly suggestive of overdiagnosis. Based on our 2 simple approaches, we estimate that somewhere between 7000 and 12 000 Taiwanese women have been overdiagnosed with lung cancer.

### Misleading Feedback: 5-Year Survival

**Figure 5.**
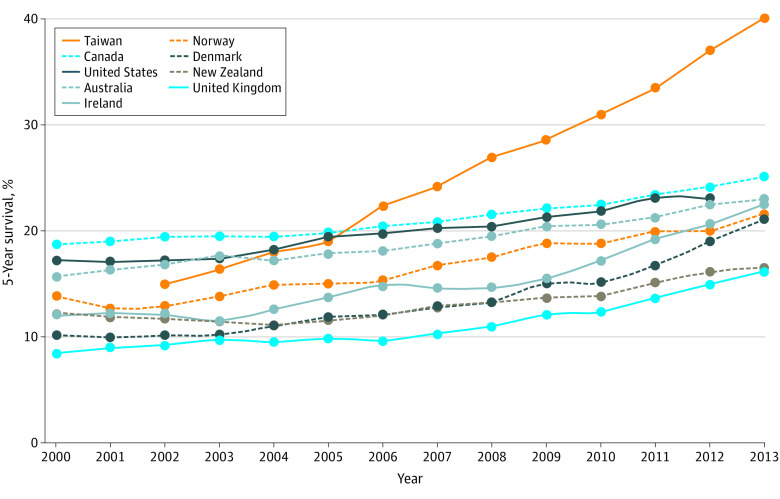
5-Year Survival Trends for Women With Lung Cancer in High-Income Countries, 2000-2013 Data are from the Cancer Survival in High-Income Countries (SURVMARK-2) project of the International Agency for Research on Cancer.^34^ The project calculates 5-year relative survival among women aged 15 to 99 years. We used matching age criteria to obtain data from Taiwan^33^ and the US.^35^

Female lung cancer mortality decreased slightly during the study period, from 17 to 16 per 100 000 population. There was a dramatic change, however, in 5-year survival after lung cancer diagnosis; it more than doubled from 18% in 2004 to 40% in 2013. There is a secular trend of modest increases in lung cancer survival among women across high-income countries, as shown in Figure 5. However, Taiwan’s experience stands out. At the start of the century, 5-year survival for Taiwanese women ranked in the middle among high-income countries; within approximately a decade, Taiwan attained what is arguably the highest lung cancer survival rate in the world.

## Discussion

Although lung cancer is typically thought of as being uniformly deadly, concerns about screening-related lung cancer overdiagnosis have been long-standing. Evidence of overdiagnosis initially appeared with the long-term follow-up of 2 randomized trials of chest radiography screening.^36,37^ The concern became more prominent after an observational study of LDCT screening reporting similar rates of lung cancer detection in smokers and nonsmokers.^38,39^ This was in stark contrast to findings by Doll and Hill^40^ that British physicians who smoked 25 or more cigarettes per day were 20 times more likely to die of lung cancer than their nonsmoking colleagues, highlighting the distinction between the risk of diagnosis and the risk of death. Consequently, both the National Lung Screening Trial^2^ and the European trial^3^ incorporated meticulously designed protocols to minimize overdiagnosis: small pulmonary nodules were followed up and underwent biopsy only if growth was established. Such growth assessment protocols, however, may not transfer well into the real world of screening practice.

To our knowledge, our findings represent the first evidence of LDCT-induced lung cancer overdiagnosis at a population level. Because the more than 6-fold increase in early-stage cancer was not accompanied with a decline in late-stage cancer, we surmise that virtually all the increased detection after the introduction of LDCT screening in 2004 represents overdiagnosis, which is considerably higher than that reported in the randomized clinical trials.^41,42^

### Alternative Explanations

Although alternative explanations to overdiagnosis are possible, they are unlikely. It is possible, for example, that the true occurrence of lung cancer is increasing in Taiwan. However, exposure to risk factors for lung cancer—smoking, second-hand smoke, and air pollution (eFigure 1 in the Supplement)—have been declining for more than 30 years. Furthermore, it is difficult to imagine how such an increase in true disease occurrence would involve solely early-stage disease. Although the prevalence of epidermal growth factor receptor mutations is highest among Asian populations,^43^ genetic drift is not a plausible explanation for changing incidence, because changes in germline inheritance require generations to emerge and, again, would be expected to affect all disease stages.

It is also possible that there has been insufficient time to see a reduction in late-stage cancer that is of similar magnitude to the increase in early-stage cancer. This explanation would require extraordinarily long lead times—at least a decade or more—well beyond the 2-year mean lead time estimated for CT.^44^

Finally, it is important to emphasize that our findings do not exclude the possibility that LDCT screening has had a small beneficial effect. Although late-stage incidence did not significantly change over the entire 2004-2018 period, a Joinpoint trend analysis (see eFigure 2 in the Supplement) suggests an small increase in late-stage disease from 2004 to 2009, followed by a small decrease in subsequent years. It is tempting to attribute the decrease to screening, but the antecedent rise is difficult to explain. Although both are marginally statistically significant, we believe neither is clinically meaningful. Nevertheless, we acknowledge that screening may have advanced the time of diagnosis of a few cancers destined to become late stage.

The small decline in mortality could be attributed to LDCT screening, as opposed to the decline in exposure to risk factors for lung cancer or to the improvements in lung cancer treatment. Regardless of whether the forgoing hypotheses are true, a possible beneficial effect of screening has no bearing on the question of whether overdiagnosis is occurring, because the two can coexist.

### Policy Context

Despite not having a claims-based measure of the primary exposure—LDCT screening for lung cancer—NHI claims do provide evidence of its downstream effects. From 2000 to 2018, claims for thoracotomies (ie, lobectomies, segmental and wedge resections) in women increased from approximately 800 to 8000 per year, virtually all reflecting the growth of video-assisted thoracoscopic surgical procedures (eFigure 3 in the Supplement). Given this evidence of both overdiagnosis and overtreatment, we would strongly encourage the NHI to continue not to provide reimbursement for population-wide LDCT screening for lung cancer. Nevertheless, these data highlight the financial incentives favoring opportunistic screening even without being a covered benefit; screening can be offered at reduced cost (even free) and generate downstream procedures that are reimbursed by the NHI.

The Taiwan experience highlights the misleading feedback that follows cancer screening. Because so few Asian women smoke, the majority of Asian women with lung cancer are nonsmokers.^45^ Screening promotion targeted this group and LDCT detected more in situ and stage I cancers. Clinicians interpret this as beneficial, because the typical patient with early-stage cancer does so well. Soon a shift in stage distribution is evident: the proportion of cancers presenting as late stage decreases—although it is simply an artifact of detecting additional early-stage cancers, not that fewer patients are presenting with late-stage cancers (eFigure 4 in the Supplement and Figure 4). Clinicians view this as a more favorable stage distribution. This view is then apparently confirmed by a substantial rise in 5-year survival, now arguably the highest in the world, despite the well-established biases associated with the measure.^46^

Although increased survival is often taken as evidence of the success of screening, survival statistics are biased by overdiagnosis.^47^ The bias is best understood by considering a simple thought experiment. At the outset, there are 100 lung cancer cases, and 5-year survival is 20% (20/100). Then lung cancer incidence increases 25% to 125 cases and all 25 new cases represent overdiagnosis. These 25 cases add to both the numerator and denominator of the survival statistic. Thus, without any change in treatment effectiveness, the resulting 5-year survival is now 36% (45/125). This thought experiment shows that the increased incidence in Taiwanese women (28% increase from 2004 to 2013) can explain virtually all the change in 5-year survival.

Survivor stories add to the misleading feedback. As more Taiwanese individuals are screened, including nonsmoking celebrities, more are found to have cancer and undergo surgery. Understandably, these survivors believe they owe their lives to screening and become its strongest supporters. Celebrity survivors have become particularly strong advocates for population-wide LDCT screening for lung cancer.^48^ Thus, the popularity paradox is at work: the more overdiagnosis and overtreatment result from screening, the more people believe they owe their life to the screening and the more popular it becomes.^49^

The misleading feedback from screening even extends to judgments about risk factors for lung cancer.^50^ For more than half a century, cigarette smoking has been known to be a powerful risk factor for death due to lung cancer.^40^ Because more never smokers are screened, however, more lung cancers are found in never smokers. At present, more than 90% of Taiwanese women diagnosed with lung cancer are never smokers.^51^ This further reinforces the idea that screening should not be restricted to heavy smokers. More concerning is that because the risk of lung cancer diagnosis is conflated with the risk of death due to lung cancer, widespread screening apparently diminishes the importance of cigarette smoking as a risk factor for lung cancer.

### Limitations

This study has some limitations. The ability to make causal inferences using observational data is always challenging and is typically limited by confounding bias.^52^ The primary limitation, however, is our inability to directly measure the exposure: use of screening LDCT. The reason is simple: LDCT screening is paid for out of pocket and is not a covered benefit of the NHI. We have data, however, showing the number of LDCT scanners increased substantially from 2003 to 2019 (from 357 to 623) and have witnessed the advent of direct-to-consumer promotion (as shown in Figure 1). Furthermore, the pattern of incidence growth—dramatically rising early-stage incidence coupled with stable late-stage incidence—is difficult to explain as anything other than an effect of screening or incidental detection.

## Conclusions

The findings of this population-based ecological cohort study suggest that LDCT screening of mostly nonsmoking Asian women was associated with considerable overdiagnosis of lung cancer. Although 2 major randomized trials^2,3^ have demonstrated reduced lung cancer mortality among heavy smokers, this benefit may be extrapolated to populations who are at lower risk for death due to lung cancer, and the data from Taiwan make clear the potential harms of this extrapolation. Until randomized trials demonstrate value to lower-risk groups, our findings suggest that LDCT screening should be offered only to heavy smokers, and only following a balanced presentation of benefits and harms^53^ (which include not only overdiagnosis but also false-positive diagnosis, more incidental findings, more diagnostic procedures, and radiation-induced cancers).^1^

Opportunistic lung cancer screening in the general population is proceeding throughout Asia, further conflating the risk of diagnosis with the risk of death. A recently published meta-analysis of 69 studies from South Korea, Japan, and China reported a pooled lung cancer detection rate of 1.12% and similar detection rates in smokers and nonsmokers.^54^ This detection rate is similar to that observed in heavy smokers randomized to the intervention group in the National Lung Screening Trial^2^ and European trial.^3^ Asian investigators are advocating screening for all adults based solely on these observations.^9,10,11,12,13,55^ Six hospitals in China have added LDCT screening to their employees’ regular health examination and have found more lung cancers in nonsmokers than smokers and in women than men, concluding the vast majority had an extremely good prognosis.^56^ Sadly, judgments about the efficacy of screening based solely on detection and survival rates are a recipe for overdiagnosis.

## Notes

Supplement. **eMethods 1.** Calculation of Stage Distribution and Stage-Specific Incidence **eMethods 2.** Estimates of the Number of Women Overdiagnosed **eFigure 1.** Improved Air Quality in Taiwan, 1990-2017 **eFigure 2.** Joinpoint Trend Analysis of Stage-Specific Female Lung Cancer Incidence, 2004-2018 **eFigure 3.** National Health Insurance (NHI Taiwan) Claims for Thoracotomies in Women, 2000-2018 **eFigure 4.** Female Lung Cancer Stage Distribution by Age, 2004-2018
