# Supplementary material for: Association of Computed Tomographic Screening Promotion With Lung Cancer Overdiagnosis Among Asian Women
Source: JAMA Intern Med. 2022 Jan 18;182(3):1–9. doi: 10.1001/jamainternmed.2021.7769 (PMC8767491; doi:10.1001/jamainternmed.2021.7769)
Supplement: Supplement. — eMethods 1. Calculation of Stage Distribution and Stage-Specific Incidence eMethods 2. Estimates of the Number of Women Overdiagnosed eFigure 1. Improved Air Quality in Taiwan, 1990-2017 eFigure 2. Joinpoint Trend Analysis of Stage-Specific Female Lung Cancer Incidence, 2004-2018 eFigure 3. National Health Insurance (NHI Taiwan) Claims for Thoracotomies in Women, 2000-2018 eFigure 4. Female Lung Cancer Stage Distribution by Age, 2004-2018 [file jamainternmed-e217769-s001.pdf]

Supplementary Online Content

Gao W, Wen CP, Wu A, Welch HG. Association of computed tomographic screening promotion with lung cancer overdiagnosis among Asian women. *JAMA Intern Med*. Published online January 18, 2022. doi:10.1001/jamainternmed.2021.7769

eMethods 1. Calculation of Stage Distribution and Stage-Specific Incidence
eMethods 2. Estimates of the Number of Women Overdiagnosed
eFigure 1. Improved Air Quality in Taiwan, 1990-2017
eFigure 2. Joinpoint Trend Analysis of Stage-Specific Female Lung Cancer Incidence, 2004-2018
eFigure 3. National Health Insurance (NHI Taiwan) Claims for Thoracotomies in Women, 2000-2018
eFigure 4. Female Lung Cancer Stage Distribution by Age, 2004-2018

This supplementary material has been provided by the authors to give readers additional information about their work.

© 2022 American Medical Association. All rights reserved.

eMethods 1. Calculation of Stage Distribution and Stage-Specific Incidence

Counts by stage & calculation of stage distribution

2004-2018 counts are from Taiwan National Cancer Registry Online Interactive Inquiry System
https://cris.hpa.gov.tw/pagepub/Home.aspx?itemNo=cr.p.20

Lung cancer in Taiwanese FEMALES												
	Counts by Stage							Early/Late Counts			Stage Distribution	
Year	0	1	2	3	4	All		Early (0-1)	Late (2-4)		Early (0-1)	Late (2-4)
2004	0	224	53	500	1254	2031		224	1807		11%	89%
2005	0	277	47	505	1359	2188		277	1911		13%	87%
2006	0	317	58	539	1484	2398		317	2081		13%	87%
2007	0	406	72	590	1728	2796		406	2390		15%	85%
2008	0	448	88	611	1764	2911		448	2463		15%	85%
2009	0	639	83	630	2096	3448		639	2809		19%	81%
2010	3	663	152	452	2227	3497		666	2831		19%	81%
2011	4	703	140	414	2275	3536		707	2829		20%	80%
2012	10	937	141	452	2450	3990		947	3043		24%	76%
2013	12	1005	151	414	2466	4048		1017	3031		25%	75%
2014	18	1276	179	408	2699	4580		1294	3286		28%	72%
2015	30	1557	167	437	2677	4868		1587	3281		33%	67%
2016	41	1806	196	439	2721	5203		1847	3356		35%	65%
2017	369	2212	195	565	2758	6099		2581	3518		42%	58%
2018	151	2544	280	464	2866	6305		2695	3610		43%	57%

© 2022 American Medical Association. All rights reserved.

Calculation of stage-specific incidence using stage distribution and overall incidence
Incidences are expressed per 100,000 and are age-adjusted to the 2000 world standard population. Incidence data are from Taiwan National Cancer Registry Online Interactive Inquiry System.
https://cris.hpa.gov.tw/pagepub/Home.aspx?itemNo=cr.p.20

Lung cancer in Taiwanese FEMALES								
		Stage Distribution			Incidence		Stage-specific Incidence	
Year		Early (0-1)	Late (2-4)		*(overall)*		Early (0-1)	Late (2-4)
		[a]	[b]		[c]		[= a x c]	[= b x c]
2004		11%	89%		21.03		2.32	18.71
2005		13%	87%		21.68		2.74	18.94
2006		13%	87%		22.37		2.96	19.41
2007		15%	85%		23.55		3.42	20.13
2008		15%	85%		23.26		3.58	19.68
2009		19%	81%		26.03		4.82	21.21
2010		19%	81%		24.97		4.76	20.21
2011		20%	80%		25.48		5.09	20.39
2012		24%	76%		27.41		6.51	20.90
2013		25%	75%		26.87		6.75	20.12
2014		28%	72%		28.72		8.11	20.61
2015		33%	67%		29.12		9.49	19.63
2016		35%	65%		30.70		10.9	19.80
2017		42%	58%		31.56		13.36	18.20
2018		43%	57%		33.69		14.40	19.29

© 2022 American Medical Association. All rights reserved.

eMethods 2. Estimates of the Number of Women Overdiagnosed

Overview

Because it is impossible to establish overdiagnosis in an individual, there is no perfect method to estimate the number of individuals overdiagnosed. Overdiagnosis is easily appreciated at a population scale, however, and it is important to provide some sense of the number of individuals affected.

On the following pages, we detail two simple approaches used to estimate the number of women overdiagnosed in Taiwan. More complex methods are, of course, possible – but they come at a cost: less transparency (the "black box" problem) and more assumptions. Whether the added complexity results in more accuracy is uncertain. Our experience is that it does not: using two more complex (and more difficult to describe) approaches produced estimates similar to those that follow.

Our simple approaches both make the same 3 assumptions:

1. There is no overdiagnosis in 2004 (and years prior).
2. There is no overdiagnosis in late-stage (Stage 2-4) lung cancer.
3. True lung cancer occurrence is stable – that is, there is no change in the underlying rate of clinically meaningful lung cancer.

Each assumption biases our estimate downwards as follows:

1. There is undoubtedly some overdiagnosis in 2004 and prior years.
2. There may be some overdiagnosis in Stage 2 lung cancer (most likely in elderly women).
3. True lung cancer occurrence is likely to be declining. While there is no change in the prevalence of smoking among females (< 5% smoking over past 40 years), it has declined sharply among males (from over 60% to less than 25%). Thus female exposure to second hand smoke has been declining – which would be expected to decrease the true occurrence of lung cancer. Furthermore, there has also been a general improvement in ambient air quality in Taiwan over the last few decades (see **eFigure 1**).

© 2022 American Medical Association. All rights reserved.

Excess early-stage incidence approach

One simple approach appears in the table below. To estimate the number of women overdiagnosed, we multiplied the absolute increase in early-stage incidence in a given year over that in observed 2004 (2.32 per 100,000) by the number of women in the population in that year. We then sum across years.

Year	Female Population	Early -stage incidence	Incidence excess	Count Overdiagnosed
	[a]	[b]	[c= b - 2.32]	[= a * c]
2004	11,147,537	2.32	0.00	0
2005	11,207,943	2.74	0.43	48
2006	11,284,820	2.96	0.64	72
2007	11,349,593	3.42	1.10	125
2008	11,410,680	3.58	1.26	144
2009	11,483,038	4.82	2.50	288
2010	11,526,898	4.76	2.44	281
2011	11,579,238	5.09	2.78	321
2012	11,642,503	6.51	4.19	487
2013	11,688,843	6.75	4.43	518
2014	11,735,782	8.11	5.79	680
2015	11,780,027	9.49	7.17	845
2016	11,820,546	10.90	8.58	1014
2017	11,851,647	13.36	11.04	1308
2018	11,876,019	14.40	12.08	1434
			**Total Overdiagnosed**	**7565**

The weakness of this approach is that early-stage incidence is age-adjusted. Taiwan is rapidly aging – from 2004 to 2017 the proportion of females age 60 or older has increased from 13% to 22%.

In an aging society, even a stable age-adjusted incidence will imply more cancer patients. Similarly, even a stable rate of cancer overdiagnosis will imply more overdiagnosed patients. Thus this approach is clearly an undercount, as is evident in the counts of cases on the following page.

© 2022 American Medical Association. All rights reserved.

Excess early-stage count approach

The table below shows the raw counts of Taiwanese women diagnosed with early-stage (Stage 0 or 1) lung cancer. Again, we assume no overdiagnosis in 2004. But because there is no decline in the incidence of late-stage lung cancer, all of the counts in excess of 2004 are taken to represent overdiagnosis.

Year	Early Stage Count	Excess Count
	[a]	[= a - 224]
2004	224	0
2005	277	53
2006	317	93
2007	406	182
2008	448	224
2009	639	415
2010	666	442
2011	707	483
2012	947	723
2013	1017	793
2014	1294	1070
2015	1587	1363
2016	1847	1623
2017	2581	2357
2018	2695	2471
**Total Overdiagnosed**		**12292**

Given the two forgoing approaches our estimate is expressed as a range: between 7 to 12 thousand Taiwanese women have been overdiagnosed.

© 2022 American Medical Association. All rights reserved.

eFigure 1. Improved Air Quality in Taiwan, 1990-2017[1]

The proportion of the Taiwanese population using solid fuel (wood, coal) dropped substantially from 36% to 5% between 1990 and 2017. Over the same period the estimated rate of death attributable to air pollution declined 40%.

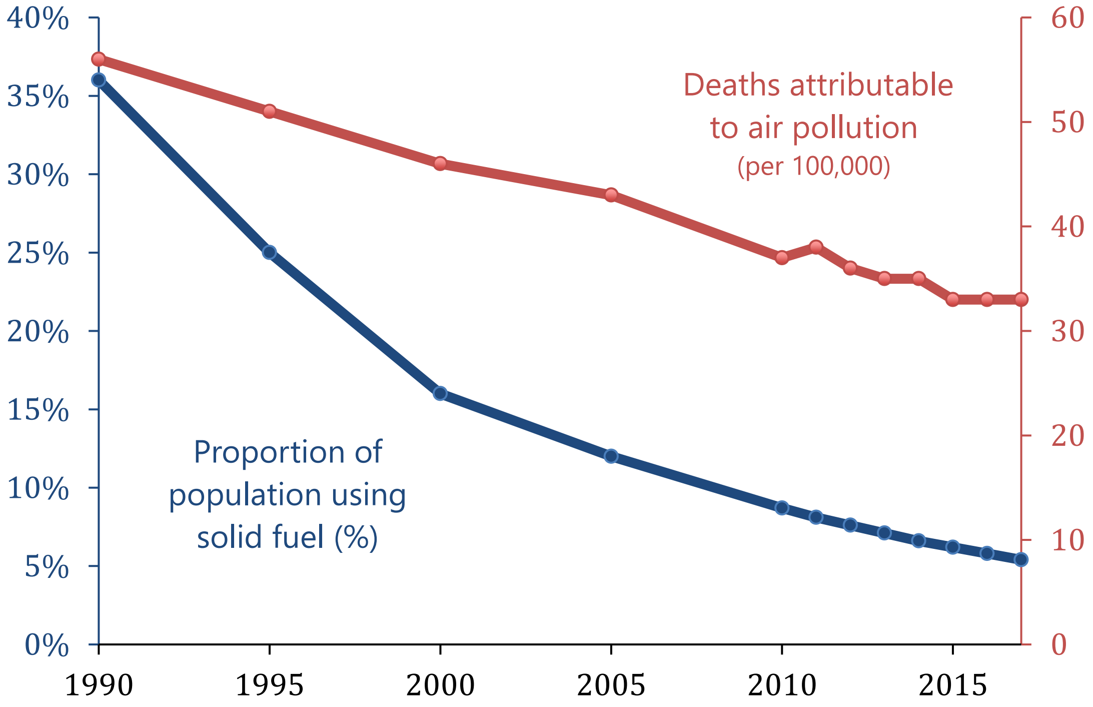

[1] Health Effects Institute. 2019. State of Global Air 2019.
https://www.stateofglobalair.org/data/#/air/plot?pollutants=pm25
https://www.stateofglobalair.org/data/#/health/plot?pollutants=airpoll

© 2022 American Medical Association. All rights reserved.

eFigure 2. Joinpoint Trend Analysis of Stage-Specific Female Lung Cancer Incidence, 2004-2018

While late-stage incidence did not significantly change over the entire period 2004-2018, a Joinpoint trend analysis detects a joinpoint (i.e. an inflection in slope) in 2009 – suggesting an increase in late-stage disease between 2004 and 2009 (APC=2.4; 95%CI: 0.5, 4.4), followed by a decrease between 2009 and 2018 (APC=-1.0; 95%CI: -1.8, -0.2).

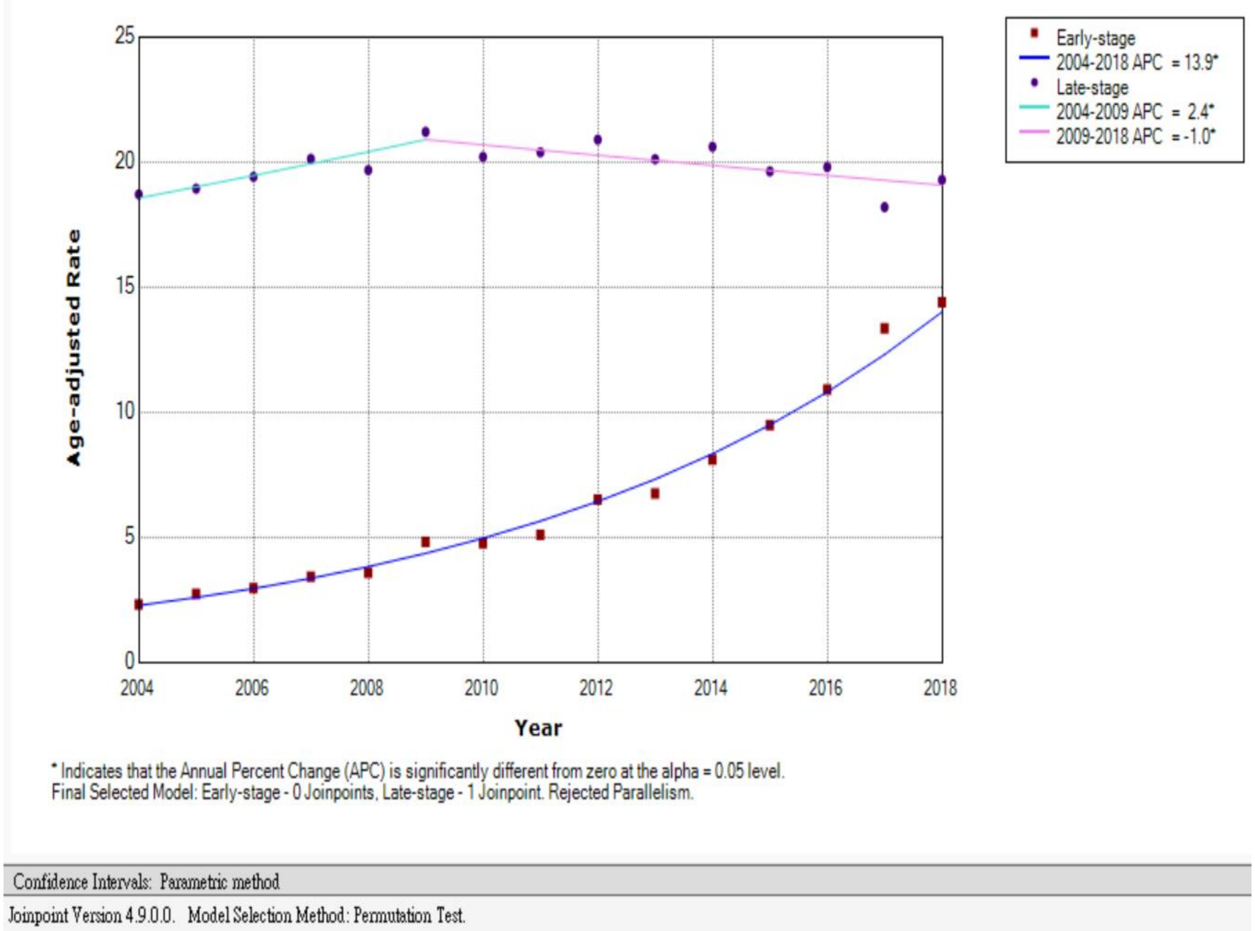

**Estimated Joinpoints**								
**Cohort**	**Joinpoint**	**Estimate**	**Lower CI**	**Upper CI**				
Late Stage	1	2009	2007	2015				
**Annual Percent Change (APC)**								
**Cohort**	**Segment**	**Lower Endpoint**	**Upper Endpoint**	**APC**	**Lower CI**	**Upper CI**	**Test Statistic (t)**	**Prob >|t|**
Early-Stage	1	2004	2018	13.9*	13.0	14.7	36.5	<0.001
Late-Stage	1	2004	2009	2.4*	0.5	4.4	2.8	0.020
Late-Stage	2	2009	2018	-1.0*	-1.8	-0.2	-2.9	0.016
*Indicates that the Annual Percent Change (APC) is significantly different from zero at the alpha = 0.05 level.								

Joinpoint Regression Program, version 4.9.0.0. https://surveillance.cancer.gov/joinpoint

Statistical Methodology and Applications Branch, S. R. P., National Cancer Institute (2021).

© 2022 American Medical Association. All rights reserved.

eFigure 3. National Health Insurance (NHI Taiwan) Claims for Thoracotomies in Women, 2000-2018

Thoracotomies include lobectomies, segmental and wedge resections of the lung.

VATS claim were introduced in 2007, all claims prior to 2007 were for conventional thoracotomies.

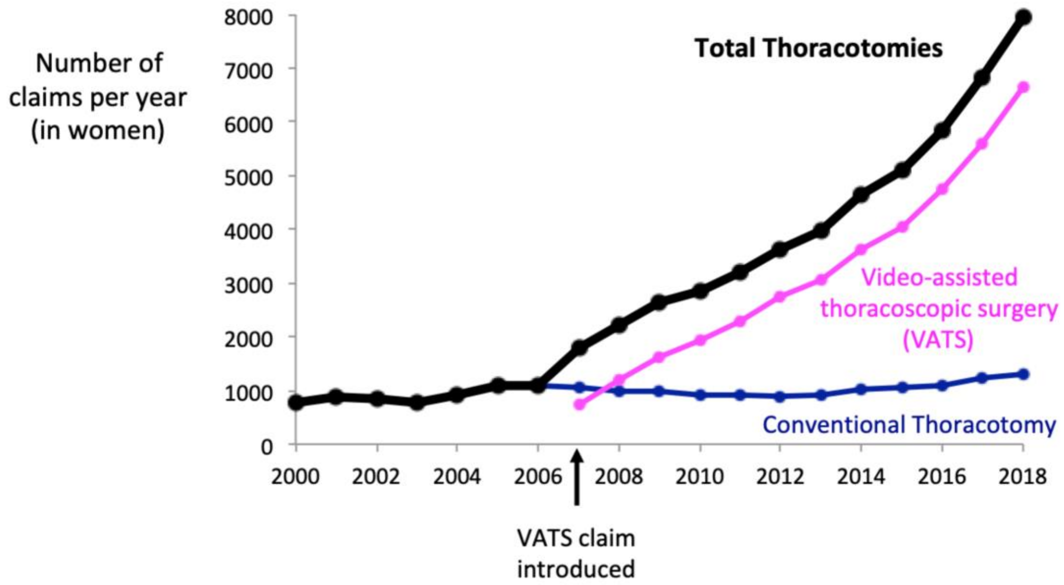

© 2022 American Medical Association. All rights reserved.

eFigure 4. Female Lung Cancer Stage Distribution by Age, 2004-2018

While late-stage incidence has been stable (see Figure 4, main paper), the additional early-stage tumors have changed the stage-distribution: the proportion of cancers presenting as late-stage has fallen. The effect is most pronounced in women under age 50, suggesting screening is disproportionately occurring in this group.

Lung Cancer stage data are from Taiwan National Cancer Registry Online Interactive Inquiry System. https://cris.hpa.gov.tw/pagepub/Home.aspx?itemNo=cr.p.20

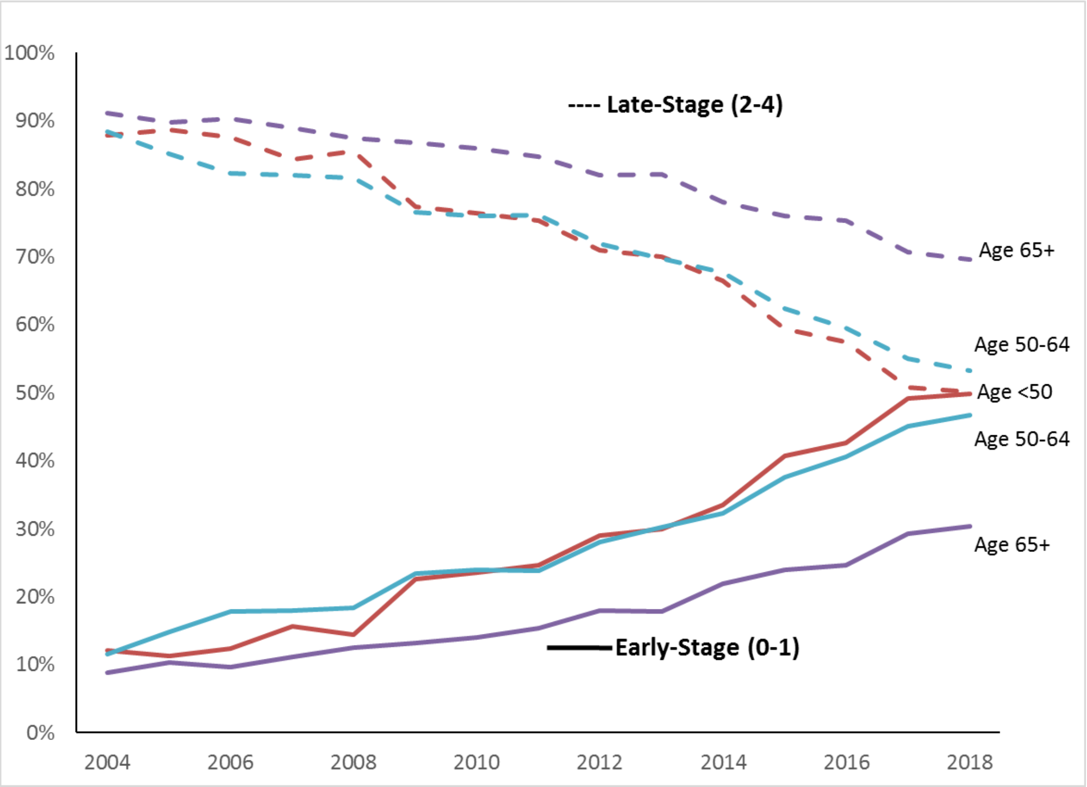

© 2022 American Medical Association. All rights reserved.
